# Molecular Dynamics and Metadynamics Simulations of the Cellulase Cel48F

**DOI:** 10.1155/2014/692738

**Published:** 2014-05-21

**Authors:** Osmair Vital de Oliveira

**Affiliations:** Federal Institute of Education, Science and Technology of Espírito Santo, Campus Vila Velha, Avenida Ministro Salgado Filho 1000, Bairro Soteco, 29106-010 Vila Velha, ES, Brazil

## Abstract

Molecular dynamics (MD) and metadynamics techniques were used to study the cellulase Cel48F-sugar. Cellulase is enzyme that breaks cellulose fibers into small sugar units and is potentially useful in second generation alcohol production. In MD simulations, the overall structure of equilibrated Cel48F did not significantly change along the trajectory, retaining root mean square deviation below 0.15 nm. A set of 15 residues interacting with the sugar chains via hydrogen bonding throughout the simulation was observed. The free energy of dissociation (ΔG_diss._) of the chains in the catalytic tunnel of Cel48F was determined by metadynamics. The ΔG_diss._ values of the chains entering and leaving the wild-type Cel48F cavity were 13.9 and 62.1 kcal/mol, respectively. We also mutated the E542 and Q543 to alanine residue and obtained ΔG_diss._ of 41.8 and 45.9 kcal/mol, respectively. These mutations were found to facilitate smooth dissociation of the sugar chain across the Cel48F tunnel. At the entry of the Cel48F tunnel, three residues were mutated to alanine: T110, T213, and L274. Contrary to the T110A-Cel48F, the mutants T213-Cel48F and L274-Cel48F prevented the sugar chain from passing across the leaving site. The present results can be a guideline in mutagenesis studies to improve processing by Cel48F.

## 1. Introduction

**Figure 1:**
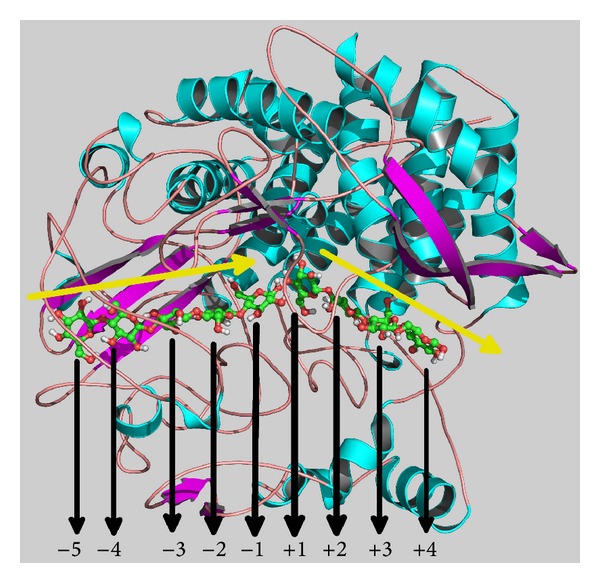
Crystallographic structure of the cellulase Cel48F interacting with two sugar chains: −5 to −1 (chain-I) and +1 to +4 (chain-II). The yellow arrows indicate the directions of the entering and the leaving sugar chains across the tunnel.

Processive enzymes are a special class of enzymes that remain attached to their polymeric substrates between multiple rounds of catalysis before dissociating from the substrates [1, 2]. Following release of the product from the active site, a new polymer chain enters the catalytic region of the enzyme. In other words, the substrate moves along the enzyme, enabling the substrate to rapidly locate the enzyme and its catalytic region. Therefore, processive activity is important for increasing enzyme efficiency. Among several processive enzymes, the cellulases have received attention as potential sources of second generation biofuel (ethanol) [3]. Cellulases catalyze the beta-1,4-glycosidic bonds of cellulose forming sugar residues, such as glucose. Subsequently, these can be fermented to alcohol or other chemicals to replace nonrenewable sources, such as petroleum and oil. Cellulose, a stable polymeric component of plant materials, is the most abundant renewable source of carbon and energy worldwide. Therefore, understanding the processive action of cellulases and identifying the role of the residues participating in the cellulase-sugar interactions can greatly benefit biofuel technologies. In the present study, we selected a processive cellulase enzyme from* Clostridium cellulolyticum*, a mesophilic anaerobic bacterium that efficiently degrades plant cell walls [4, 5]. This bacterium secretes 36 cellulosomal proteins, the majority of which are glycosidic hydrolases belonging to families 5, 8, 9, 10, 11, 18, 26, 27, 44, 48, and 74 in the CAZy database (http://www.cazy.org/) [6]. Among the most abundant cellulases in the cellulosomes of* C. cellulolyticum* are the endoprocessive cellulases Cel48F (family 48), by which the bacterium digests cellulose [7, 8]. Cel48F is composed of two modules: an N-terminal catalytic module and a C-terminal docking module, which attaches the enzyme to the scaffolding protein [8, 9]. The Cel48F active site, located in the catalytic domain, comprises a long closed tunnel (length 2.5 nm) followed by an open cleft region [10], as shown in Figure 1. This structural characteristic is common to family 48 cellulases from other organisms. Cellulases of this family are characterized by liberation of a cellobiose moiety by a processive mechanism [10–12]. In addition, they hydrolyze cellulose via a single displacement mechanism, which inverts the anomeric carbon configuration [13]. According to previous reports, the acidic and catalytic amino acid is the E55 residue [10, 14]. Mutating this glutamic acid residue to glutamine inactivates the Cel48F enzyme. On the other hand, the basic catalytic residue is not well defined, although residues E44 and D230 have been suggested to play proton donor roles [15]. Cel48F enzymes in which E44 has been replaced with glutamine retain around 1% of their former activity [14]. Saharay et al. [16] conducted computational studies to determine the enzymatic mechanism of another family 48 enzyme (CelS), with high structural similarity to Cel48F, which also contains residues E44 and D230. These authors identified the D255 residue, corresponding to D230 in Cel48F, as the most probable base catalyst, but their results are not conclusive. This controversy was finally resolved by Kostylev and colleagues [17], who showed that the Cel48A catalytic base in* Thermobifida fusca*, a thermophilic soil bacterium, is D225 (equivalent to D230 in* C. cellulolyticum* Cel48F). However, the processivity mechanism and the roles of the residues in the catalytic tunnel of family 48 cellulases remain unclear. Therefore, in the present study, we used computational techniques to investigate the processive action of Cel48F and its interactions with sugar chains in aqueous solution.

## 2. Material and Method

### 2.1. Standard Molecular Dynamics

The goal of this standard MD simulation is to equilibrate the Cel48F-sugar chains complex so as to be used posteriorly in metadynamics simulations. The Cel48F-sugar chains complex was obtained from the Protein Data Bank (PDB ID 1F9D) [10]. In this complex, the sugar chain positions are subdivided into two sections: a tunnel section ranging from position −5 to −1 (representing the entered group) and an open cleft section from position +1 to +4 (representing the leaving group); see Figure 1. This nomenclature was proposed by Davies et al. [18]. Moreover, in this crystallographic structure, the residue E55 was mutated to Q55. Therefore, to obtain the native Cel48F, we replaced the residue Q55 to E55. The Cel48F-sugar chain complex was inserted into a box with edges 9.0 nm, filled by 21,047 water molecules. To maintain the system electrostatically neutral, eight sodium ions were added by replacing preexisting water molecules. The energy of the system was minimized by sequentially applying the steepest [19] and conjugated gradient [20] algorithms, yielding an energy gradient below 200 kJmol^−1^. The solvent molecules were allowed to relax for 300 ps, while the Cel48F-sugar chains complex was rigidly retained by imposing a positional restraint potential (force constant 1000 kJmol^−1 ^nm^−2^). The positional restraints were then removed, and the system was equilibrated for 5 ns. The molecular dynamics (MD) simulations were conducted using the GROMOS 53A6 force field [21] for protein and sugar chains, while the water molecules and sodium ions were described by the simple point charge model [22] and the Chandrasekhar parameters [23], respectively. The calculations were performed using the GROMACS 4.0.7 program [24, 25]. The simulation was conducted in the NpT ensemble using the Berendsen thermostat [26] (at 300 K and relaxation time 0.1 ps). The pressure was 1 bar (with compressibility 4.5 × 10^−5^ bar^−1^ and relaxation time 1 ps). Periodic boundary conditions were imposed with a 1.0 nm cutoff for nonbonded interactions. Long-range electrostatic corrections were modeled by the particle mesh Ewald technique [27, 28]. The equations of motion were integrated by the leapfrog algorithm [29] with an integration step of 1.0 fs.

### 2.2. Metadynamics

The sugar chains entering and leaving the Cel48F tunnel (chain-I and chain-II, resp.) were investigated by metadynamics, a technique for efficiently computing free energies and for accelerating rare events [30, 31]. In this method, a set *s* of collection variables (CVs), which are functions of the system coordinate *x*, must be well defined to represent an intrinsic characteristic of the system, for example, the distance between two molecules. The bias potential is represented by the following equation:

(1) V(s,t)=∑ti=(Δt,2Δt,3Δt,…)ti<twexp⁡((s−s(ti))22δs2),

where Δ*t* is the time interval between two successive Gaussians, *δs* is the Gaussian width, and *w* is the Gaussian height. These are free parameters that mediate the efficiency and accuracy of the algorithm; hence, they must be chosen carefully. In this study, we set Δ*t* = 0.1 ps, *δs* = 0.05 nm, and *w* = 0.5 kJ·mol^−1^. Metadynamics were performed in two sequential steps: (1) leaving of sugar chain-II (Figure 1), on the basis of a snapshot obtained at 5 ns of the previous standard MD simulation and (2) entry of sugar chain-I along the cellulase cavity (Figure 1). The CV in step 1 was distance between the centers of mass of Cel48F and sugar chain-II, and simulation was terminated when the Cel48F-sugar chain-II interactions reached zero. Before metadynamics to be applied in step 2, 1.0 ns of standard MD simulation was performed in the final system (when chain-II had been liberated from the Cel48F tunnel) of step 1. Metadynamics simulation was then performed using a snapshot at 1.0 ns sampled from the prior MD simulation. Similar to step 1, the CVs of step 2 were the centers of mass of Cel48F, and the simulation continued until a cellobiose had located at the cleavage site (the O-glycosidic bond of chain-I locates between E55 and E44 or at the D230 residue of Cel48F). To ensure smooth leaving and entry of chains II and I, respectively, some residues were mutated to alanine. These new systems were equilibrated for 500 ps in a standard MD simulation. Subsequently, metadynamics simulations were performed on the basis of a snapshot at 500 ps. All metadynamics simulations were carried out using the algorithm proposed by Laio and Parrinello [30], implemented in the PLUMED plugin [32] coupled to the GROMACS computational package.

## 3. Results and Discussion

### 3.1. Standard Molecular Dynamics

**Figure 2:**
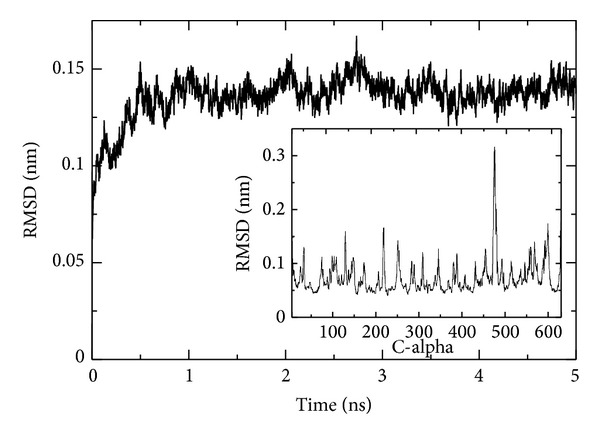
RMSD of the C*α* atoms in the entire Cel48F molecule as a function of the simulation time. Inset shows the RMSF of the C*α* atoms.

This section describes the results from the equilibrated 5 ns of standard MD simulation of the Cel48F-sugar chains complex in an aqueous solution. Although this simulation time is short, it is sufficient to equilibrate the system (see Figure 2). Therefore, this equilibrium simulation was used to predict the free energy using metadynamics technique in the next section.

Figure 2 shows the root mean square deviation (RMSD) of the C*α* atoms in the entire enzyme Cel48F. The RMSD is below ~0.15 nm, implying that the enzyme undergoes no significant structural change, compared with the initial structure (0 ns), throughout the 5 ns MD time course. The RMSD value increases rapidly from 0 to 1 ns and then fluctuates around a value of ~0.14 nm after ~1 ns, suggesting that the system reached an equilibration after 1 ns. Furthermore, the position of the sugar chains in the enzyme tunnel, obtained at the end of the simulation time, is very similar to its initial position (at 0 ns) keeping RMSD value of 0.01 nm with standard deviation of 0.001 nm along the simulation. Therefore, the small structural change induced in Cel48F by the aqueous media does not affect the position of the sugar chains in the tunnel.

In addition, the fluctuation in the C-alpha atoms is low (RMSF values around 0.1 nm), except at residues 474–477 (Figure 2, inset). These residues form part of the E466-L484 loop, 3.5 nm distant from the catalytic tunnel. The low RMSF values for E55 (0.06 nm), E44 (0.04 nm), and D230 (0.05 nm) reflect the low mobility of these residues; consequently, their positions are conserved along the MD trajectory.

**Figure 3:**
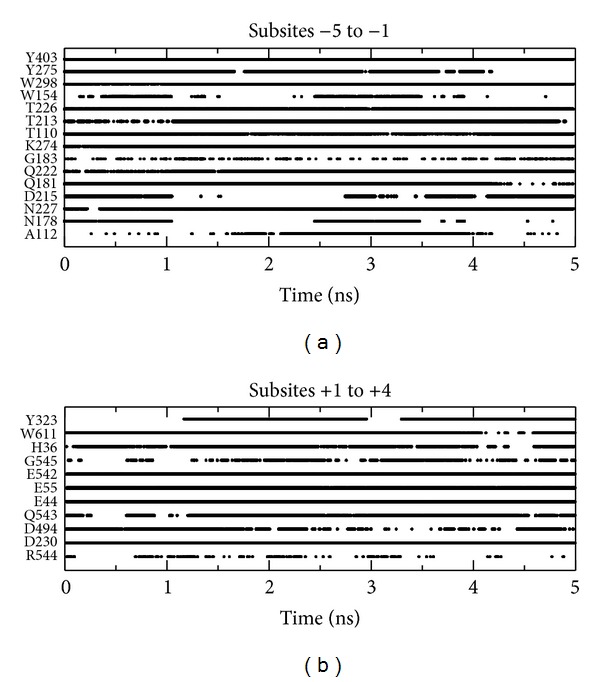
Hydrogen bonds between enzyme and sugar chains as a function of the simulation time.

Hydrogen bond interactions are extremely important in biological systems, for example, in enzymes, where they are responsible for ligand stabilization. In this way, we have measured the hydrogen bonding based on two geometric criteria: (1) the distance between the hydrogen donor and the acceptor atoms must be shorter than 0.35 nm and (2) the angle formed by the donor, hydrogen donor, and acceptor atoms must exceed 120°.  Figure 3 displays all residues that form hydrogen bond with the sugar chains during the 5 ns MD time course.

**Figure 4:**
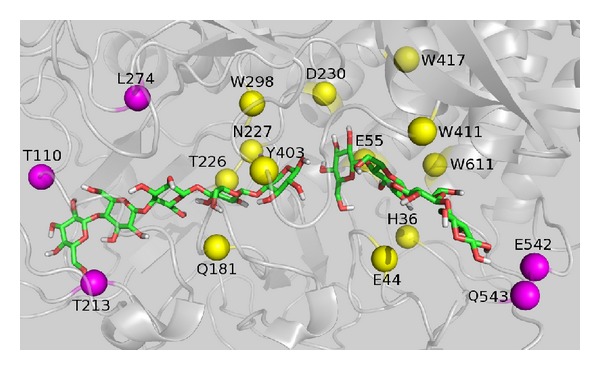
Snapshot at 5 ns showing the C*α* atoms (spheres) of the most frequently hydrogen-bonded residues within Cel48F. By the yellow and magenta colors are represented the residues candidate to be mutated and the residues that cannot be mutated, respectively. The residues W411 and W417 are presented by its importance in the aromatic contact with the sugar chain. For clarity, the apolar hydrogen atoms in the sugar chains are not shown.

In Figure 3, the time intervals during which hydrogen bonds form and break throughout the 5 ns MD simulation are clearly visible. In Figure 4 is presented the snapshot in the end of MD simulation highlighting the residues that form stable hydrogen bonds, except W411 and W417, with the sugar chains.

Analyzing Figures 3 and 4, we observe stable hydrogen bond formation between the sugar chains and 15 Cel48F residues: N227, Q181, K274, T110, T213, T226, W298, Y403, D230, E44, E55, E542, H36, Q543, and W611. Except for T213, T226, and D230, this set corresponds to the residues observed in crystallographic structure of the enzyme. Therefore, along the simulation, the residues T213, T226, and D230 are reorientated to stabilize the sugar chain into the enzyme tunnel.

With few exceptions, all of the residues presented in Figure 4 were found to be conserved in three other family 48 cellulases:* Clostridium thermocellum* (PDB code: 1LY1) [32],* Anaerocellum thermophilum* (PDB code: 4EL8) [33], and* Hahella chejuensis* (PDB code: 4FUS) [34], where each cellulase is identified by its PDB code. The exceptions are residues A577, K547, and K583 in 1L1Y, 4EL8, and 4FUS, respectively, corresponding to residue Q543 in Cel48F. Residue D223 in 4EL8 corresponds to N277 in Cel48F, while S136 in 4FUS corresponds to T110 in Cel48F. Residue I210 in 4EL8 corresponds to T213 in Cel48F. These investigations reveal that the residues shown in Figure 4 have a large degree of conservation throughout the family 48 cellulases, suggesting their importance in the stabilization of the sugar chains via hydrogen bonding.

At subsites +1 to +4, the hydrogen bond analysis reveals stable interactions formed by residues D230, E44, E55, E542, H36, Q543, and W611 of cellulase Cel48F (Figure 3). Therefore, these residues are responsible for stabilizing the sugar chain within the Cel48F tunnel. As a consequence, for the leaving of chain-II, this must occur by rupture of these interactions. Interestingly, residues E55 (the acid catalyst) and D230 and E44 (both likely candidates for the base catalyst) interact with the sugar chain, implying that these residues help to stabilize the chain-II after it has participated in enzymatic activity. Given the importance of these residues in the enzymatic mechanism [10], it cannot be used in mutagenesis studies to facilitate the leaving of chain-II. In detail, the side chain of E55 is positioned between subsites +1 and +2, while the side chain of E44 points toward subsites +2/+3. Finally, residue D230 interacted by hydrogen bonds with subsite +1. Along the simulation, residue H36 was observed to form hydrogen bond with both E55 and sugar chain-II (in 30% of the MD trajectory; Figure 3), suggesting two roles for H36: to stabilize the leaving group and to increase the pKa of E55. Some studies have shown the importance and the role of histidine in glycoside hydrolases [35–39]. In the crystallographic structure of the Cel48F, the distance of the H36 with E55 and subsite +1 is far, with distance of 0.45 and 0.34 nm, respectively. Therefore, the results from MD simulation show clearly the importance of the solvent effect in the H36-E55 and H36-sugar chain interactions. Although H36 stabilizes chain-II, mutating this residue would remove its ability to increase the pKa of E55. This increase is attributed to the hydrogen network and electrostatic effects [36]. According to literature reports, aromatic residues act as lubricating agents to reduce the sliding barrier in processive action [10]; consequently, in our understanding the residue W611 cannot be mutated. Although aromatic residues W411 and W417 do not form hydrogen bonds with chain-II, they are in contact with this chain along the 5 ns of MD simulation. For instance, subsites +1 and +2 are stabilized by W417 via hydrophobic interactions keeping distance of 0.82 nm with standard deviation (sd) of 0.03 nm between centers of mass along the simulation. While the subsite +3 is stabilized by W411 via a possible pi-stacking interaction, keeping distance of 0.49 nm with sd of 0.03 nm between centers of mass, which must be confirmed and quantified by quantum method. Thus, among the residues forming the most hydrogen bonds with chain-II, residues E542 and Q543 alone (shown as magenta spheres in Figure 4) are suitable for mutagenesis study. These residues are positioned in a loop (N531-D546) at the end of the Cel48F tunnel. Therefore, the flexibility of this loop plays an important role in the leaving of chain-II. Mutating these residues to alanine, which has a small side chain, should yield a higher flexibility of the loop, inducing smooth leaving of chain-II through the Cel48F tunnel. In the next section of this study, these residues were mutated to alanine, and the free energy of dissociation (ΔG_diss._) of Cel48F-sugar chain-II was calculated.

From Figures 3 and 4, we observe that the following residues form stable hydrogen bonds between Cel48F and chain-I: N227, Q181, K274, T110, T213, T226, W298, and Y403. Residues Q181, T226, N227, W298, and Y403 form a circle around subsites −2 and −1 of chain-I (Figure 4). We believe that this arrangement or network of hydrogen bonds guides the sugar chain into an ideal position for enzymatic processing; therefore, in our understanding, these residues should not be targeted in mutagenesis studies. Interestingly, subunit −1 is stabilized by hydrogen bonds rather than by hydrophobic interactions with W298 and Y403. Therefore, among the above listed residues, we mutate T110, T213, and K274 to alanine and compute the ΔG_diss._ of the sugar chain-I entering across the mutated Ce48F. For instance, the residues T110 and T213 interact with subsites −5 and −4, and both are located at the end of the tunnel (magenta spheres in Figure 4) and K274 interacts with subsite −3 (Figure 4).

### 3.2. Metadynamics Simulations

**Figure 5:**
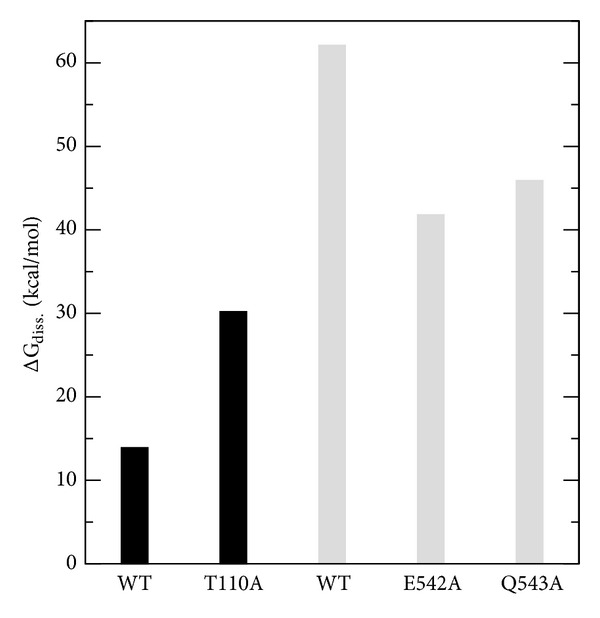
Free energy of dissociation for the wild-type (WT) and mutated Cel48F enzymes obtained from metadynamics. Black and gray bars indicate residues that interact with the entering (chain-I) and leaving (chain-II) sugar chains, respectively.

Due to the computational cost, the standard MD simulations cannot operate over the long time scales required for the sugar chains to pass through the Cel48F tunnel. This limitation can be overcome by metadynamics using a bias potential between two molecules, for example, distance between the center of mass of the enzyme and the sugar chain. Metadynamics was performed in two steps: the leave of sugar chain-II, followed by the entry of a cellobiose unit from sugar chain-I through the cellulase Cel48F tunnel. Furthermore, ΔG_diss._ was calculated for those residues that were mutated to alanine (indicated by magenta spheres in Figure 4).  Figure 5 shows the ΔG_diss._ of the successful mutations obtained in the metadynamics calculations. Although the L274 and T213 were mutated to alanine, they does not improve the processivity, as explicated below. Therefore, these mutations are not shown in Figure 5.

Metadynamics was used to predict the dissociation free energy of WT-Cel48F, E542A-Cel48F, and Q543A-Cel48F complexed with sugar chains. Since these residues interact with the leaving group (chain-II), this technique essentially forces chain-II to leave the cellulase cavity. Following catalysis, this is the first step that occurs at experimental level or in live organisms. In all free energy calculations the error was 1-2 kcal/mol. The following ΔG_diss._ values (Figure 5) were obtained: 62.1, 45.9, and 41.8 kcal/mol for WT-Cel48F, Q543A-Cel48F, and E542A-Cel48F, respectively. Therefore, the free energy of association for each glucose unit in the WT-Cel48F is −15.5 kcal/mol. This value was obtained by the following approximation: dividing the total free energy of association (−62.1 kcal/mol) by four (which correspond to the four glucose units). This value is, reasonably, in agreement with that the obtained by Bu et al. [40] using the Cel7A from* Trichoderma reesei*. This cellulase, like here, has a closed tunnel and the free energy of binding of a glucose residue using the steered molecular dynamics is −10.9 kcal/mol [40]. Although Cel7A and Cel48F have a closed tunnel, there are significant structural differences among these. Therefore, the free energy of binding will be expected to be different, but these two values are in the same order of magnitude. The small ΔG_diss._ values of the mutated residues (relative to WT) obtained here are attributable to the nonformation of hydrogen bonds between the alanine residues and the sugar chain. Because association is contrary to dissociation process, the binding free energy of chain-II is the negative of the above determined ΔG_diss._. The ΔG_diss._ values clearly demonstrate that mutations of Q543 and E542 to alanine facilitate the smooth passing of chain-II across the Cel48F tunnel. In other words, these mutations can accelerate product expulsion from the Cel48F tunnel. The results above are a guide for experimental mutagenesis studies.

After the chain-II has leaved the Cel48F tunnel (step 1), we performed 1 ns of standard MD simulation to equilibrate the configuration obtained from the metadynamics simulation of step 1. This procedure was necessary because the final configuration was not in thermodynamic equilibrium. This new simulation yielded very low RMSD (0.08 nm for all C*α* in the Cel48F), indicating that the overall structure of the enzyme is stable during simulation. In addition, the final position of chain-I in the active site is similar to that at the initial simulation time. The snapshot obtained in the last time (1 ns) of the standard MD trajectory was used in the metadynamics simulation. Here we have used as a collective variable the distance between the center of mass of the enzyme and chain-I. For the entry process (step 2), two criteria were used to stop the metadynamics calculations: (i) a cellobiose had passed through the catalytic cavity and (ii) its O-glycosidic bond had positioned between D230 (catalytic acid) and E44 and E55 (the likely catalytic base candidates). Under these criteria, the calculated ΔG_diss._ values (Figure 5) were 13.9 and 30.2 kcal/mol in WT-Cel48F and T110A-Cel48F, respectively. Clearly, the mutation of T110 to alanine does not improve the insertion of sugar chain into the cleavage site. On the other hand, when L274 and T213 are replaced with alanine, the sugar chain-I passes contrary to the cleavage site, which is unfavorable to processivity. On the basis of our results, we propose that our procedure (combined standard MD and metadynamics technique) can identify suitable candidate residues for mutagenesis studies for other cellulases.

## 4. Conclusions

Given the importance of cellulase enzymes in converting cellulose to small sugar units, we investigated the cellulase Cel48F-sugar chains complex in aqueous solution using standard MD simulations and metadynamics. The overall structure of cellulase Cel48F was observed to be stable throughout 5 ns of standard MD simulation. During this time interval, we identified a set of 15 residues (N227, Q181, K274, T110, T213, T226, W298, Y403, D230, E44, E55, E542, H36, Q543, and W611) that form stable interactions via hydrogen bonding with the sugar chains. Among these, we selected suitable residues for mutation to alanine, aiming to obtain a low energetic barrier for the leave and entry of sugar chains through the catalytic Cel48F tunnel. For the leaving sugar chain, the ΔG_diss._ values obtained from metadynamics were 62.1, 45.9, and 41.8 kcal/mol for WT-Cel48F, Q543A-Cel48F, and E542A-Cel48F, respectively. For the entering sugar chain, the ΔG_diss._ values were 13.9 and 30.2 kcal/mol for WT-Cel48F and T110A-Cel48F, respectively, revealing that mutation of T110 to alanine is unfavorable for processivity process. The present results and conclusions can be applicable to future mutagenesis studies.
